# Effect of body dissatisfaction on binge eating behavior of Chinese university students: A moderated mediation model

**DOI:** 10.3389/fpsyg.2022.995301

**Published:** 2022-11-03

**Authors:** Jimin Yan, Haodong Su, Chunlu Li

**Affiliations:** ^1^Department of Psychology, School of Medical Humanitarians, Guizhou Medical University, Guiyang, China; ^2^School of Humanities and Social Sciences, Binzhou Medical University, Yantai, China; ^3^Guizhou Health Development Research Center, Guiyang, China

**Keywords:** body dissatisfaction, perceived stress, binge eating behavior, self-acceptance, cognitive reappraisal, expressive suppression

## Abstract

The relationship between body dissatisfaction and binge eating behavior has been highlighted by previous studies. However, the psychological mechanisms underlying body dissatisfaction-induced binge eating behavior remain unclear. Here, we further addressed this issue in the framework of the sociocultural model of eating disorders. Firstly, we investigated the mediation effect of perceived stress on the relationship between body dissatisfaction and binge eating. Secondly, we examined the moderation role of the self-acceptance and emotion regulation strategies on the indirect effect of body dissatisfaction on binge eating behavior mediated by perceived stress. Data from 903 Chinese university students were analyzed using SPSS 26.0 and SPSS PROCESS Macro. Results indicated that perceived stress mediates the relationship between body dissatisfaction and binge eating behavior. Main interactional effects have been observed when self-acceptance and cognitive reappraisal but not expressive suppression are introduced in the model as a moderator. Implications and limitations of the study are discussed.

## Introduction

Binge eating behavior is defined as excessive caloric intake coupled with a sense of loss of control over the eating. It is a trans diagnostic characteristic of several eating disorders (EDs), including binge eating disorder (BED), bulimia nervosa (BN), and anorexia nervosa binge-purge subtype (American Psychiatric Association, 2013). Binge eating behavior has numerous psychiatric and medical comorbidities, such as dental caries, acute gastric dilatation, gastric perforation (Sato and Fukudo, 2015) and non-suicidal self-injury (Pak et al., 2021). Furthurmore, it is a serious condition often associated with lower quality of life, functional impairment in work, home or personal life (Pak et al., 2021). The lifetime prevalence of BED is estimated to be approximately 3% with more prevalence in women in the United States (Hudson et al., 2007). Emerging adulthood, roughly ages 18–25 (Arnett, 2000, 2010), appears to be high risk for the development and persistence of binge eating behavior (Goldschmidt et al., 2015). Consistently, it was found that college students, most in emerging adulthood, are more likely to appear binge eating behavior than middle school students in China (Shiyu, 2020). A recent study found 85 cases of binge eating disorder among 1,103 Chinese college students (Yan et al., 2018). Given its high prevalence and severe negative consequences, the causes and maintenance of binge eating in Chinese college students need to be fully understood. Such work would lead to the development of effective and affordable treatments for binge eating behavior.

Numerous studies have documented the powerful role of sociocultural factors on the development and maintenance of eating and body image disorders (Jackson and Chen, 2007; Weissman, 2019). Today’s media and sociocultural espouses that thinness is more attractive and can lead to social rewards such as acceptance and happiness (Twamley and Davis, 1999). Many women aspire to these ideals, but this is almost impossible to achieve in reality, leading to body dissatisfaction among women (Twamley and Davis, 1999). Therefore, the relationship between body dissatisfaction and binge eating behavior has received increasing attention. Body dissatisfaction is one of the most robust predictors to eating disorders (Lenza, 2020) and clinically negative body image has been a central diagnostic feature for all eating disorders (Justine et al., 2015). However, body dissatisfaction does not always result in binge eating. Therefore, it is necessary and valuable to distinguish the factors that may interact with body dissatisfaction in predicting binge eating.

According to the sociocultural model of eating disorders, those who internalize media-espoused thin ideal are more likely to experience body dissatisfaction, a perceived discrepancy between their appearance and the thin ideal. In turn, body dissatisfaction might lead to more pressure to be thin and more efforts to lose weight/dietary restraint, and finally results in subsequent eating disorders. In fact, perceived stress has been shown to be a significant contributor to binge eating disorder (Striegel-Moore et al., 2007; Richardson et al., 2015; Thurston et al., 2018; Ohara et al., 2019; Smith et al., 2020). That is to say, it seems that body dissatisfaction leads to binge eating behaviors mediated by perceived stress, including social comparison, appraisals of potential positive regard from thinness and the pressure to be thin. This hypothesis is supported by a plenty of evidence in the sample from western developed countries (Shomaker and Furman, 2009; Bailey and Ricciardelli, 2010; Webb and Zimmer-Gembeck, 2014). On the other hand, a recent study found that although the prevalence of body dissatisfaction was higher in youth from a low socioeconomic status (SES), while body dissatisfaction did not predict increased risk for binge eating behavior in this population; on the contrary, body dissatisfaction predicted increased risk for binge eating behavior among adolescents from high SES backgrounds (West et al., 2019). It appears that the associations between body dissatisfaction and binge eating disorder may be moderated by SES. Thus, the psychological mechanisms underlying the relationship between body dissatisfaction and binge eating behavior might differ among youth from different SES. China is a developing country and different with western developed countries in terms of socioeconomic status. It remains unclear whether this hypothesis is also supported by the Chinese sample. Therefore, here we tested the aforementioned hypothesis in Chinese sample: perceived stress mediates the relationship between body dissatisfaction and binge eating behavior (H1).

Self-acceptance refers to an individual’s acceptance of all of his/her attributes, positive or negative (Morgado et al., 2014), and is a central characteristic of psychological health, optimal functioning and maturity (Ryff and Singer, 1996). The individual’s sense of self-acceptance has been found to be negatively associated with perceived stress (Xu et al., 2017; Sun et al., 2019). For example, numerous studies of mindfulness revealed that greater person’s self-acceptance can reduce perceived stress (Rodriguez et al., 2015; Sun et al., 2019). Higher self-acceptance leads to lower pressure to change, since that true self-acceptance is accepting yourself without conditions or exceptions (Seltzer, 2008). Thus, in the context of body dissatisfaction, individuals with high self-acceptance were expected to perceive less stress, and conversely, individuals with low self-acceptance were expected to perceive more stress. Therefore, we hypothesized that self-acceptance moderates the relationship between body dissatisfaction and perceived stress (H2).

Negative emotions have been found to be an important mediator of the relationship between perceived stress and eating disorders in young Chinese women (Chen et al., 2012). Some studies have found that binge eating behavior maintains *via* negative reinforcement, since it serves to reduce momentary negative emotional states (Wonderlich et al., 2010). Further, emotion dysregulation is one of risk and vulnerability factors for individuals who engage in binge eating behavior (Claes and Muehlenkamp, 2014; Cucchi et al., 2016). The relationship between emotion dysregulation and eating pathology had consistently been found, especially in the situation experiencing strong negative emotions (Farstad et al., 2016; Farstad and Ranson, 2020). On the other hand, college students in emerging adulthood are struggling to hone their emotion regulation abilities, and may be at risk for mental health problems (LeBlanc et al., 2020). Thus, it is reasonable to hypothesize that emotion regulation strategies moderate the relationship between perceived stress and binge eating behavior in Chinese college students.

According to the process model of emotion regulation proposed by Gross (Gross, 2001), emotion may be regulated at five points in the emotion generative process: selection of the situation, modification of the situation, deployment of attention, change of cognitions (cognitive reappraisal), modulation of experiential, behavioral, or physiological responses (expressive suppression). Of these strategies, cognitive reappraisal and expressive inhibition are the two most commonly used in everyday life and the easiest to clearly define in terms of individual differences (Gross and John, 2003). Therefore, we selected these two strategies in the present research, and supposed that cognitive reappraisal (H3) and expressive suppression (H4) moderate the relationship between perceived stress and binge eating behavior, respectively. Cognitive reappraisal is conceptualized as the attempt to reinterpret an emotion-eliciting situation in a way that changes the meaning of the situation and alters its emotional impact without changing it objectively. It is an antecedent-focused emotion regulation strategy, occurring early before the emotional response trend is fully established. While expressive suppression is a reaction-centered strategy, which appears relatively in the late stages of emotion generation and primarily alters the behavioral aspects of emotional reactivity (Gross and John, 2003).

**Figure 1:**
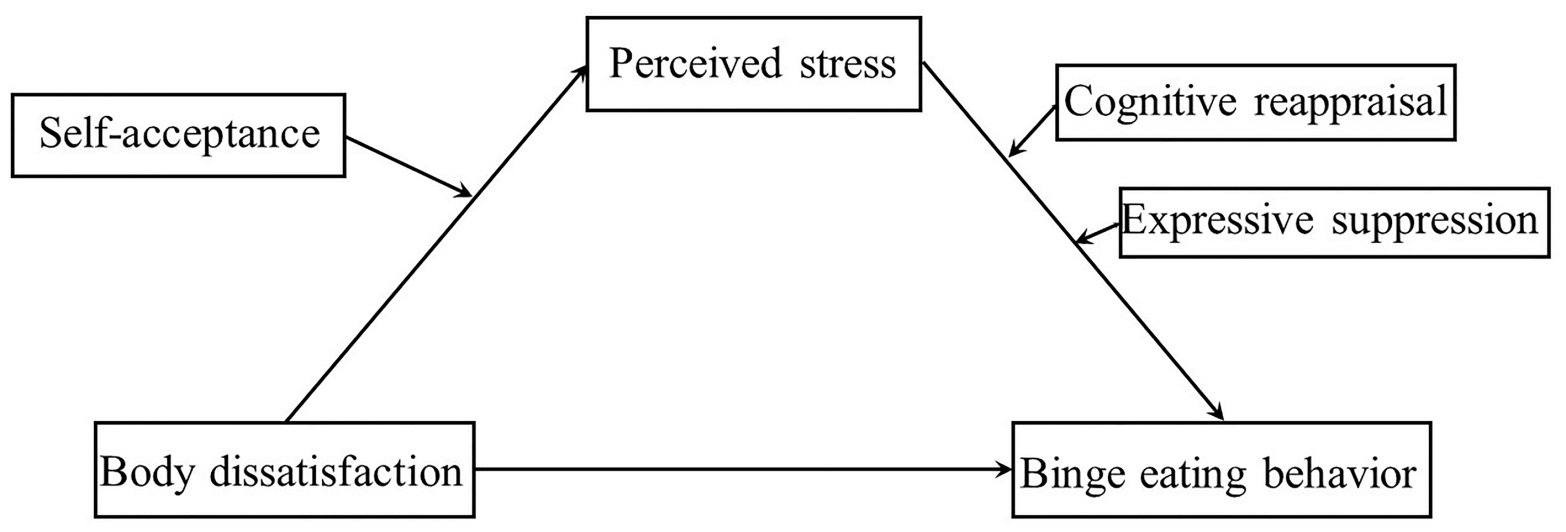
Research model.

Together, we aim to examine these four following hypotheses (as shown in Figure 1):

*H1*: perceived stress mediates the relationship between body dissatisfaction and binge eating behavior.

*H2*: self-acceptance moderates the relationship between body dissatisfaction and perceived stress.

*H3*: cognitive reappraisal moderates the relationship between perceived stress and binge eating behavior.

*H4*: expressive suppression moderates the relationship between perceived stress and binge eating behavior.

## Materials and methods

### Participants and procedures

This research was approved by the ethics committee of Guizhou Medical University. Chinese college students aged 18-25 were selected from six universities in China. At the beginning of the survey, participants were told their responses would be anonymous and they could withdraw from participation at any time, all of them signed informed consent forms. Then, a pencil and paper survey were given in a classroom. During the assessment, participants were encouraged to answer each question carefully and independently. After the evaluation, the questionnaire was withdrawn on the spot and each participant received a snack worth about 1 Yuan RMB. To ensure data quality, we excluded the data from two subject groups: (1) Those who might select the same answer for equal or greater than half the length of the total scale (Curran, 2016); (2) Those who are not within the stage of emerging adulthood, older than 25 or younger than 18. Finally, data from 903 participants (298 male and 605 female) with an age range of 18–25 years (*M* = 19.88, SD = 1.164) were included in the analysis.

### Measures

#### Cosmetic mental state self-scale

Body dissatisfaction was measured with scale I in the self-rating scale of aesthetic mental state compiled by Zhou et al. (2000). The scale contains five items (e.g., “I am ashamed of my appearance and have a low appearance rating.”), uses on a 5-Likert point scale, 1 = almost never, 5 = almost always. The sum of the scores for each item serves as an indicator of body dissatisfaction. The Cronbach’s coefficient in this study was 0.828.

#### Emotion regulation questionnaire

To measure participants’ emotion regulation strategy, the emotion regulation questionnaire compiled by Gross and John (2003) and revised by Wang et al. (2007) was used in this study. The scale includes two dimensions: cognitive reappraisal (e.g., I control my emotions by changing the way I think about the situation I’m in) and expressive suppression (e.g., I control my emotions by not expressing them.). The items of each dimension are summed separately (Gross and John, 2003). The scale has 10 items and uses a 7-Likert point scale, 1 = almost never, 7 = almost always. In the current study, Cronbach’s alpha was 0.804.

#### The self-acceptance questionnaire

To measure the self-acceptance, the Self-Acceptance questionnaire (SAQ) developed by Cong and Gao (1999) was used. SAQ includes two dimensions: self-acceptance (e.g., I’m always afraid to do things for fear of not doing well.) and self-evaluation (e.g., Overall, I am satisfied with myself.), with 8 items in each dimension. The items of self-acceptance were reverse scoring. The scale was scored on a 4-Likert point scale, total score for each item was used to reflect the self-acceptance of participants. In the current study, Cronbach’s alpha was 0.805.

#### Perceived stress scale

To measure the perceived stress, the Chinese version of Perceived Stress Scale (PSS) revised by Tingzhong Yang (Yang and Huang, 2003) was used. PSS is developed by Cohen et al. (1983) and includes two dimensions: tension (e.g., In the last month, how often have you been upset because of something that happened unexpectedly.) and sense of loss of control (e.g., In the last month, how often have you dealt successfully with irritating life hassles.). Each of them contains 7 items. The items of tension were scored in the reverse direction. The scale was scored on a 5-Likert point scale, 1 = almost never, 5 = almost always. The scores of each item were summed as the index for the perceived stress. In the present study, Cronbach’s alpha was 0.873.

#### Binge eating scale

To measure binge eating behavior, BES developed by Gormally was used (Gormally et al., 1982). This scale has 16 items self-report questionnaire, with a total score ranging from 0 to 46. In our study, the Chinese version of the BES (He et al., 2014) was used. In the current study, Cronbach’s alpha was 0.879.

### Statistical analysis

Data collected in the present study were processed using SPSS 26.0, according to Hayes (2017) described in his book. First of all, we calculated the descriptive statistics and correlations between variables. Then, by using PROCESS macro Model 4, we examined the mediation effect of perceived stress. Third, we used PROCESS macro Model 1 to examine the moderation role of self-acceptance, cognitive reappraisal and expressive suppression, respectively. Finally, we ran PROCESS macro Model 21 to examine simultaneously all the study hypotheses, as presented in Figure 1. Data were calculated based on standardized scores. Bootstrapped confidence interval (CI; 5,000 bootstrap samples) for the indirect effect was obtained. If zero is not included in the confidence interval, effects are significant.

## Results

### Preliminary analyses

Table 1 shows the descriptive statistics and correlations for the variables in the present study. Body dissatisfaction is positively associated with perceived stress (*r* = 0.46, *p* < 0.01) and binge eating behavior (*r* = 0.44, *p* < 0.01). Perceived stress is positively associated with binge eating behavior (*r* = 0.43, *p* < 0.01). Self-acceptance is negatively associated with body dissatisfaction (*r* = −0.50, *p* < 0.01) and perceived stress (*r* = −0.60, *p* < 0.01). Cognitive reappraisal is negatively associated with perceived stress (*r* = −0.40, *p* < 0.01) and binge eating behavior (*r* = −0.22, *p* < 0.01); while, expressive suppression is positively associated with perceived stress (*r* = 0.13, *p* < 0.01), and binge eating behavior (*r* = 0.11, *p* < 0.01).

**Table 1:** Descriptive statistics and correlations.

	*M*	SD	1	2	3	4	5	6
1. BD	4	2.88	–					
2. PS	24.38	6.57	0.46^**^	–				
3. BEB	9.86	8.01	0.44^**^	0.43^**^	–			
4. SA	40.53	6.23	−0.50^**^	−0.60^**^	−0.39^**^	–		
5. CR	29.09	5.42	−0.17^**^	−0.40^**^	−0.22^**^	0.34^**^	–	
6. ES	15.52	4.33	0.19^**^	0.13^**^	0.11^**^	−0.13^**^	0.19^**^	–

N = 903. BD, Body dissatisfaction; PS, Perceived stress; CR, Cognitive reappraisal; ES, Expressive suppression; SA, Self-acceptance; BEB, Binge eating behavior. ^**^p < 0.01.

### Testing for mediation effect

We ran PROCESS macro Model 4 to test hypothesis 1. The specifications of this model can be seen in Table 2. After controlling for gender, grade and major, the mediator and dependent variable models show that body dissatisfaction was significantly associated with binge eating behavior (*β* = 0.30, *p* < 0.001. see Model 2 of Table 2) and perceived stress (*β* = 0.45, *p* < 0.001. see Model 1 of Table 2). And the relationship between perceived stress and binge eating behavior was also significant (*β* = 0.29, *p* < 0.001. see Model 2 of Table 2). The resampling procedure indicates a significant indirect effect since the CI at 95% does not include the value of zero (as shown in Table 2). Therefore, perceived stress mediated the relationship between body dissatisfaction and binge eating behavior, supporting Hypothesis 1.

**Table 2:** Testing the mediation effect of perceived stress on binge eating behavior.

Predictor	Model 1 (Perceived stress)				Model 2 (Binge eating behavior)			
	*β*	*t*	*SE*	95%CI	*β*	*t*	*SE*	95%CI
Constant	−0.28	−1.55	0.18	−0.62 ~ 0.07	−0.39	−2.25^*^	0.17	−0.73 ~ −0.05
Gender	0.10	1.62	0.06	−0.02 ~ 0.23	0.09	1.53	0.06	−0.03 ~ 0.21
Grade	0.02	0.67	0.04	−0.05 ~ 0.10	0.07	1.82	0.04	−0.01 ~ 0.14
Major	0.03	0.63	0.04	−0.06 ~ 0.11	0.05	1.27	0.04	−0.03 ~ 0.14
BD	0.45	15.10^***^	0.03	0.39 ~ 0.51	0.30	9.23^***^	0.03	0.24 ~ 0.36
PS					0.29	8.85^***^	0.03	0.22 ~ 0.35
*R^2^*	0.21				0.26			
*F*	60.24				63.44			

N = 903. The Beta values are standardized coefficients, thus they can be compared to determine the relative strength of different variables in the model. Each column is a regression model that predicts the criterion at the top of the column. BD, Body dissatisfaction; PS, Perceived stress. ^*^p < 0.05, ^***^p < 0.001.

### Testing for the moderation effect

Self-acceptance is expected to moderate the indirect association between body dissatisfaction and perceived stress in the H2. To examine this moderation effect, we drew on the PROCESS macro model1. Results was shown in Table 3. The interaction between body dissatisfaction and self-acceptance was significant (*β* = − 0.08, *p* < 0.001, see Model 3 of Table 3), so the relationship between body dissatisfaction and perceived stress was moderated by self-acceptance, supporting Hypothesis 2.

**Table 3:** Testing the moderation effect of self-acceptance, cognitive reappraisal and expressive suppression.

Predictors	Model 3 (Perceived stress)				Model 4 (Binge eating behavior)				Model 5 (Binge eating behavior)			
	*β*	*t*	*SE*	95%CI	*β*	*t*	SE	95%CI	*β*	*t*	SE	95%CI
Constant	−0.07	−0.48	0.16	−0.38 ~ 0.23	−0.37	−2.06^*^	0.18	−0.72 ~ −0.02	−0.42	−2.30^*^	0.18	−0.77 ~ −0.06
Gender	0.05	0.85	0.06	−0.06 ~ 0.16	0.13	1.94	0.06	−0.00 ~ 0.25	0.14	2.21^*^	0.07	0.02 ~ 0.27
Grade	0.03	0.88	0.03	−0.03 ~ 0.10	0.07	1.75	0.04	−0.01 ~ 0.14	0.08	2.02^*^	0.04	0.00 ~ 0.15
Major	−0.04	−1.01	0.04	−0.11 ~ 0.03	0.01	0.26	0.04	−0.07 ~ 0.09	0.02	0.47	0.04	−0.06 ~ 0.10
BD	0.18	5.93^***^	0.03	0.12 ~ 0.24								
SA	−0.51	−16.73^***^	0.03	−0.56 ~ −0.45								
BD × SA	−0.08	−3.47^***^	0.02	−0.12 ~ −0.03								
PS					0.39	11.97^***^	0.03	0.33 ~ 0.46	0.41	13.30^***^	0.03	0.35 ~ 0.47
CR					−0.06	−1.67	0.03	−0.12 ~ 0.01				
PS × CR					−0.06	−2.57^*^	0.02	−0.11 ~ −0.02				
ES									0.06	1.97^*^	0.03	0.00 ~ 0.12
PS × ES									0.03	1.21	0.03	−0.02 ~ 0.08
*R^2^*	0.40				0.20				0.20			
*F*	100.15				37.33				36.38			

N = 903. The Beta values are standardized coefficients, thus they can be compared to determine the relative strength of different variables in the model. Each column is a regression model that predicts the criterion at the top of the column. BD, Body dissatisfaction; PS, Perceived stress; SA, Self-acceptance; CR, Cognitive reappraisal; ES, Expressive Suppression. ^*^p < 0.05, ^***^p < 0.001.

Cognitive reappraisal and expressive suppression are expected to moderate the indirect association between perceived stress and binge eating behavior in H3 and H4, respectively. Similarly, we ran PROCESS macro model1 to test their moderating effects. Results was also shown in Table 3. The interaction between perceived stress and cognitive reappraisal was significant (*β* = −0.06, *p* < 0.05, see Model 4 of Table 3), supporting H3 that cognitive reappraisal moderated the relationship between perceived stress and binge eating behavior. However, the interaction between perceived stress and expressive suppression was not significant (*β* = 0.03, *p* > 0.05, see Model 5 of Table 3), therefore, H4 was not supported by our data.

**Figure 2:**
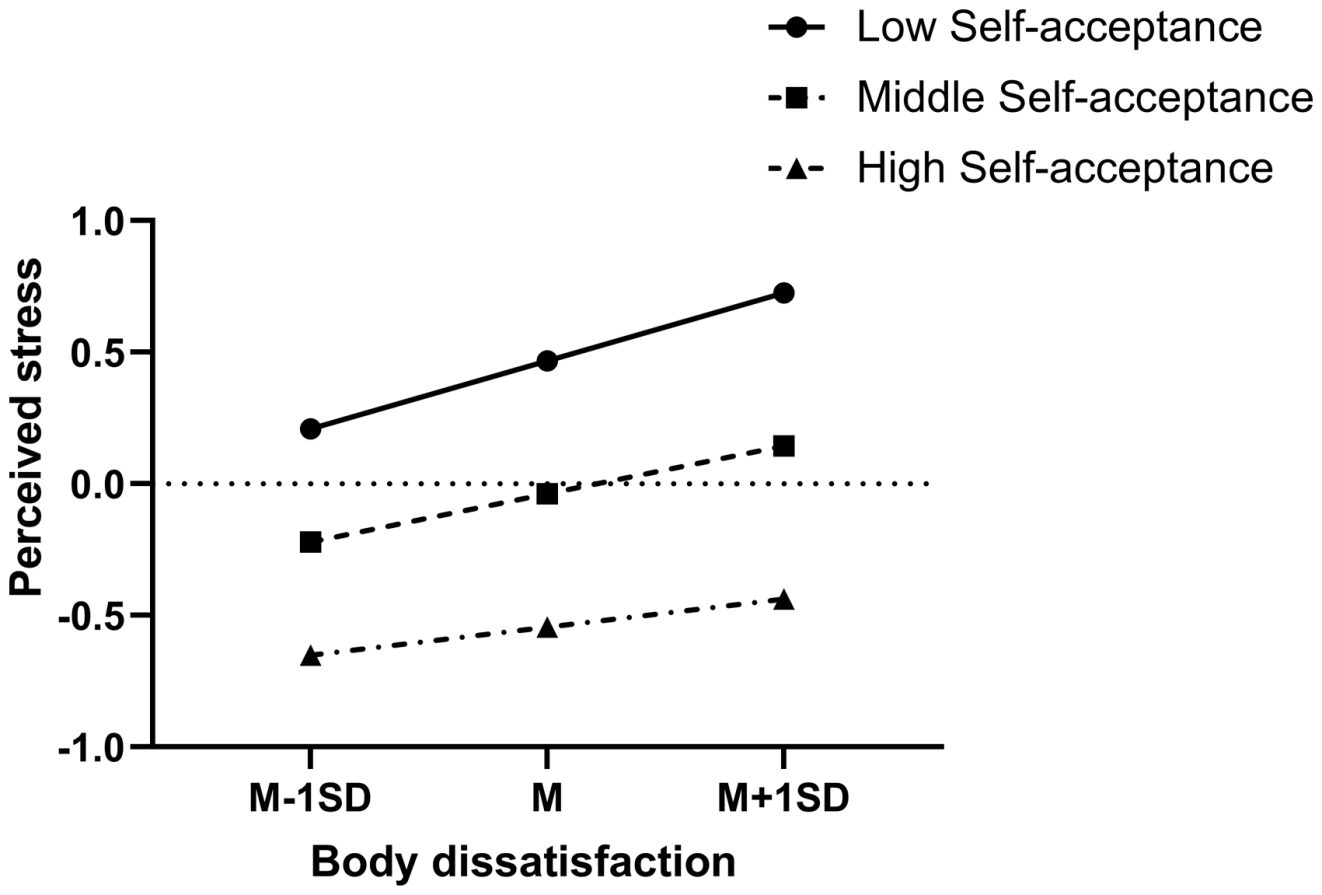
Interaction effect of body dissatisfaction and self-acceptance on perceived stress. High and low levels of body dissatisfaction and self-acceptance represent one standard deviation above and below the mean, respectively.

To describe it more directly, figure of predicted perceived stress against body dissatisfaction was plotted. Low and high levels of self-acceptance (1 SD below the mean and 1 SD above the mean, respectively) were showed in the figure separately (Figure 2). Result suggested that body dissatisfaction was significantly associated with perceived stress for both highly and lowly agreeable self-acceptance (*b*_high self-acceptance_ = 0.11, *p* < 0.01; *b*_low self-acceptance_ = 0.26, *p* < 0.001), but participants with high level of self-acceptance showed a lower level of perceived stress (see Figure 2). It is notable that highly self-acceptance adolescents were less likely to be influenced by body dissatisfaction compared with lowly self-acceptance ones (*b*_high self-acceptance_ = 0.11, *p* < 0.01; *b*_low self-acceptance_ = 0.25, *p* < 0.001).

**Figure 3:**
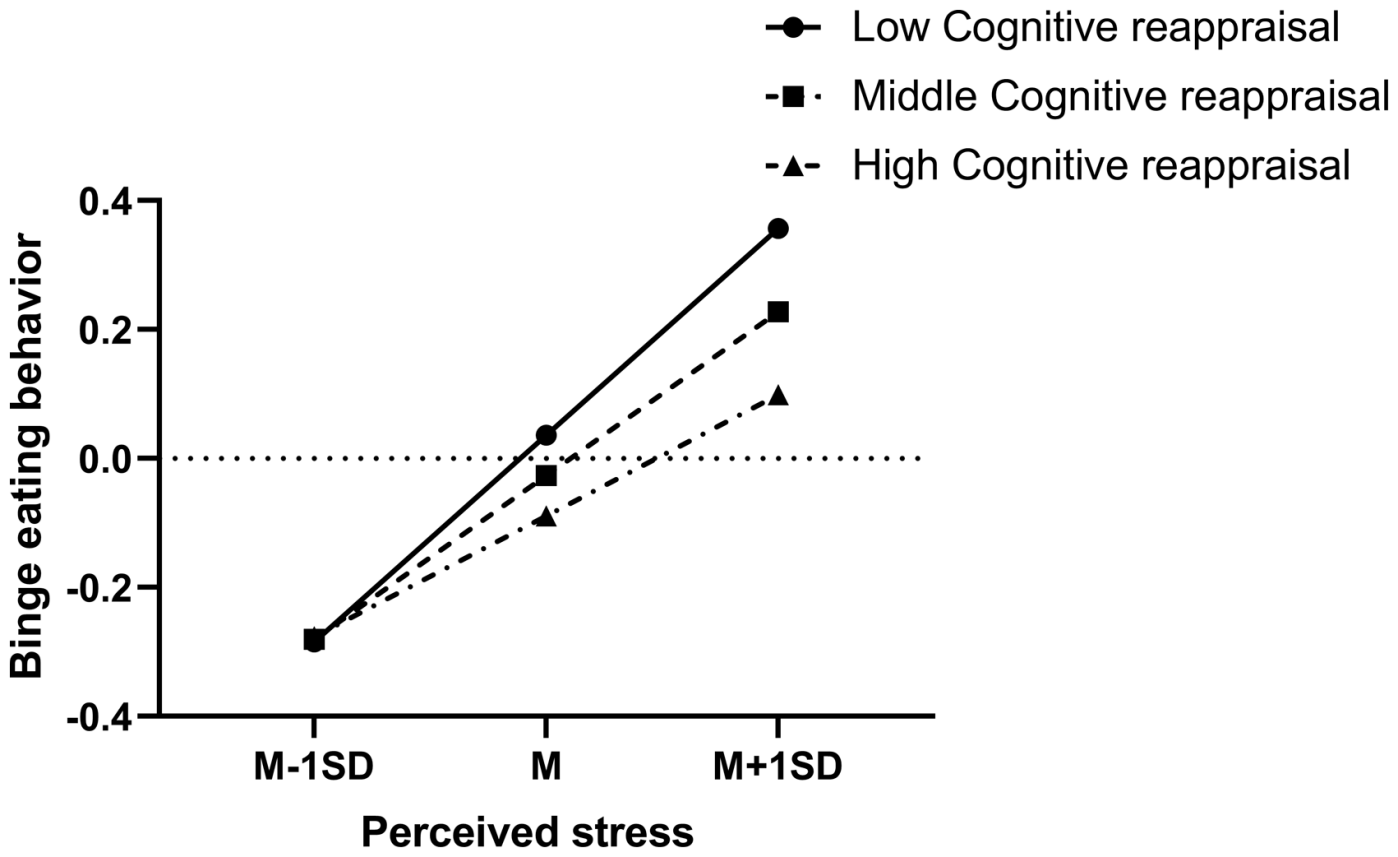
Interaction effect of perceived stress and cognitive reappraisal on binge eating behavior. High and low levels of perceived stress and cognitive reappraisal represent one standard deviation above and below the mean, respectively.

The relationship between perceived stress and binge eating behavior for low and high level of cognitive reappraisal was also plotted (see Figure 3). High levels of perceived stress were significantly associated with high levels of binge eating behavior for both highly and lowly cognitive reappraisal adolescents (*b*_high cognitive reappraisal_ = 0.19, *p* < 0.001; *b*_low cognitive reappraisal_ = 0.32, *p* < 0.001), but the binge eating behavior level was lower for those highly cognitive reappraisal participants with high level of perceived stress (see Figure 3). To be specific, highly cognitive reappraisal participants were less easily to become binge eating behavior compared with lowly cognitive reappraisal ones (*b*_high cognitive reappraisal_ = 0.19, *p* < 0.001; *b*_low cognitive reappraisal_ = 0.32, *p* < 0.001).

### Testing for the whole research model

Finally, we tested simultaneously all the study hypotheses, using PROCESS macro model 21. The result was presented in Table 4. Results of the bias-corrected percentile bootstrap method showed that the relationship between body dissatisfaction and binge eating behavior was mediated by perceived stress. And the indirect effect of body dissatisfaction on binge eating behavior *via* perceived stress was moderated by self-acceptance (*β* = −0.08, *p* < 0.001. see Model 6 of Table 4) and cognitive reappraisal (*β* = −0.07, *p* < 0.01. see Model 7 of Table 4). The index of moderated moderated mediation was.0050 with a 95% confidence interval (CI) of [0.0001, 0.0105] (see Model 8 of Table 4). Therefore, the whole research model was supported.

**Table 4:** Testing for the research model.

Predictors	Model 6 (Perceived stress)				Model 7 (Binge eating behavior)			
	*β*	*t*	*SE*	95%CI	*β*	*t*	*SE*	95%CI
Constant	−0.07	−0.48	0.16	−0.38 ~ 0.23	−0.37	−2.16^*^	0.17	−0.71 ~ −0.03
Gender	0.05	0.85	0.06	−0.06 ~ 0.15	0.09	1.53	0.06	−0.03 ~ 0.22
Grade	0.03	0.87	0.03	−0.03 ~ 0.09	0.05	1.39	0.04	−0.02 ~ 0.12
Major	−0.04	−1.01	0.04	−0.11 ~ 0.03	0.04	1.08	0.04	−0.04 ~ 0.12
BD	0.18	5.93^***^	0.03	0.12 ~ 0.24	0.30	9.37^***^	0.03	0.24 ~ 0.37
SA	−0.51	−17.73^***^	0.03	−0.56 ~ −0.45				
BD × SA	−0.08	−3.47^***^	0.02	−0.12 ~ −0.03				
PS					0.25	7.34^***^	0.03	0.19 ~ 0.32
CR					−0.06	−1.97^*^	0.03	−0.12 ~ −0.00
PS × CR					−0.07	−2.83^**^	0.02	−0.11 ~ −0.02
*R^2^*	0.40				0.27			
*F*	100.15				47.65			
Model 8								
Index of moderated moderated mediation		*β*			*SE*	95%CI		
		0.0050			0.0027	0.0001–0.0105		

N = 903. The Beta values are standardized coefficients, thus they can be compared to determine the relative strength of different variables in the model. Each column is a regression model that predicts the criterion at the top of the column. BD, Body dissatisfaction; PS, Perceived stress; SA, Self-acceptance; CR, Cognitive reappraisal. ^*^p < 0.05. ^**^p < 0.01. ^***^p < 0.001.

## Discussion

The relationship between body dissatisfaction and binge eating behavior has been highlighted by numerous studies (Justine et al., 2015; Lenza, 2020). However, the psychological mechanisms underlying body dissatisfaction-induced binge eating behavior remain unclear. Here, we further addressed this issue in the framework of the sociocultural model of eating disorders (Holland and Tiggemann, 2016). First of all, we tested the mediation effect of perceived stress on the relationship between body dissatisfaction and binge eating. Then we examined the moderation role of self-acceptance and emotion regulation strategies on the indirect effect of body dissatisfaction on binge eating behavior mediated by perceived stress.

Firstly, in the framework of the sociocultural model of eating disorders, perceived stress was expected to mediate the relationship between body dissatisfaction and binge eating behavior. To be specific, the individuals with body dissatisfaction were expected to experience more perceived stress from social comparison and the pressure to be thin, which ultimately leads to binge eating behaviors. In the present research, perceived stress was showed to partially mediate the relationship between body dissatisfaction and binge eating behavior. Therefore, the aforementioned hypothesis was also supported in Chinese sample.

A recent study suggested the associations between body dissatisfaction and binge eating disorder may be moderated by SES (West et al., 2019); and China, as a developing country, has a lower SES, compared with developed western countries. These evidences suggest that the psychological mechanisms underlying the relationship between body dissatisfaction and binge eating behavior are different in China and in Western countries. However, our data did not support this hypothesis, suggesting that the relationship between body dissatisfaction and binge eating behavior also was mediated by perceived stress in Chinese sample.

Secondly, we identified a moderation role of self-acceptance on the relationship between body dissatisfaction and perceived stress. With the same level of physical dissatisfaction, individuals with lower levels of self-acceptance experience more perceived stress. In other words, increasing self-acceptance can reduce perceived stress in physically dissatisfied individuals. Self-acceptance, especially unconditional self-acceptance, has been shown to be an effective way to reduce perceived stress in a variety of emotional and behavioral disorders, such as lower self-esteem (Chamberlain and Haaga, 2001), depressive symptoms (Chamberlain and Haaga, 2001) and job burnout (Hill et al., 2008; Sun et al., 2019). Consistently, our results suggest that it might be a potentially effective intervention to improve the self-acceptance for body dissatisfaction-induced binge eating.

Finally, this study explores the moderation effect of emotion regulation strategies on the indirect effect of body dissatisfaction on binge eating behavior mediated by perceived stress. In the present study, we found that cognitive reappraisal, but not expressive suppression moderates the indirect effect of body dissatisfaction on binge eating behavior mediated by perceived stress. As shown in Figure 3, at low levels of perceived stress, the level of cognitive reappraisal did not affect binge eating behavior; while at high levels of perceived stress, highly cognitive reappraisal participants were less easily to become binge eating behavior compared with lowly cognitive reappraisal ones. In the present study, it is suggested that body dissatisfaction-induced binge eating behavior might be effectively relieved though improving cognitive reappraisal levels.

On the contrary, expression suppression is often expected to exacerbate stress-induced binge-eating behavior. However, our results did not support this hypothesis in the context of body dissatisfaction-induced binge eating behavior.

This study makes several contributions. At a theoretical level, it improves our understanding of the mechanisms of body dissatisfaction-induced binge eating. Body dissatisfaction might lead to binge eating behavior mediated by perceived stress from one’s social environment. The self-acceptance and cognitive reappraisal but not expressive suppression might moderate the indirect effect of body dissatisfaction on binge eating behavior mediated by perceived stress. At a practical level, our results in the present suggested that the self-acceptance and cognitive reappraisal might be potentially effective interventions to ameliorate the body dissatisfaction-induced binge eating, although further experiments are still needed.

The present study had some limitations. First, it was a cross-sectional design so that we cannot draw causal conclusions. Future longitudinal studies are still needed to further confirm these conclusions. Second, college students, a relatively easy-to-obtain sample, was used in this study, so it is not known whether these conclusions can be generalized to non-college students in the stage of emerging adulthood.

## Conclusion

Body dissatisfaction leads to binge eating behavior mediated by perceived stress. Self-acceptance and cognitive reappraisal but not expressive suppression moderate the indirect effect of body dissatisfaction on binge eating behavior mediated by perceived stress.

## Data availability statement

The raw data supporting the conclusions of this article will be made available by the authors, without undue reservation.

## Ethics statement

The studies involving human participants were reviewed and approved by the Ethics Committee of Guizhou Medical University. The participants provided their written informed consent to participate in this study.

## Author contributions

JY, HS, and CL designed the study. JY and HS performed the statistical analysis. JY wrote the first draft of the manuscript. CL revised it critically for important intellectual content. All authors contributed to the article and approved the submitted version.

## Funding

This research was funded by the Guizhou Medical University start-up fund for doctoral talent (J-[2021]050).

## Conflict of interest

The authors declare that the research was conducted in the absence of any commercial or financial relationships that could be construed as a potential conflict of interest. The authors declare no conflict of interest.

## Publisher’s note

All claims expressed in this article are solely those of the authors and do not necessarily represent those of their affiliated organizations, or those of the publisher, the editors and the reviewers. Any product that may be evaluated in this article, or claim that may be made by its manufacturer, is not guaranteed or endorsed by the publisher.
